# Development and Application of Transcriptome-Derived Microsatellites in *Actinidia eriantha* (Actinidiaceae)

**DOI:** 10.3389/fpls.2017.01383

**Published:** 2017-08-25

**Authors:** Rui Guo, Jacob B. Landis, Michael J. Moore, Aiping Meng, Shuguang Jian, Xiaohong Yao, Hengchang Wang

**Affiliations:** ^1^Key Laboratory of Plant Germplasm Enhancement and Specialty Agriculture, Wuhan Botanical Garden, Chinese Academy of Sciences Wuhan, China; ^2^College of Life Sciences, University of Chinese Academy of Sciences Beijing, China; ^3^Department of Botany and Plant Sciences, University of California, Riverside Riverside, CA, United States; ^4^Department of Biology, Oberlin College Oberlin, OH, United States; ^5^South China Botanical Garden, Chinese Academy of Sciences Guangzhou, China

**Keywords:** *Actinidia eriantha*, high-throughput sequencing, transcriptome, EST-SSRs, population genetic structure

## Abstract

*Actinidia eriantha* Benth. is a diploid perennial woody vine native to China and is recognized as a valuable species for commercial kiwifruit improvement with high levels of ascorbic acid as well as having been used in traditional Chinese medicine. Due to the lack of genomic resources for the species, microsatellite markers for population genetics studies are scarce. In this study, RNASeq was conducted on fruit tissue of *A. eriantha*, yielding 5,678,129 reads with a total output of 3.41 Gb. *De novo* assembly yielded 69,783 non-redundant unigenes (41.3 Mb), of which 21,730 were annotated using protein databases. A total of 8,658 EST-SSR loci were identified in 7,495 unigene sequences, for which primer pairs were successfully designed for 3,842 loci (44.4%). Among these, 183 primer pairs were assayed for PCR amplification, yielding 69 with detectable polymorphism in *A. eriantha*. Additionally, 61 of the 69 polymorphic loci could be successfully amplified in at least one other *Actinidia* species. Of these, 14 polymorphic loci (mean *N*_*A*_ = 6.07 ± 2.30) were randomly selected for assessing levels of genetic diversity and population structure within *A. eriantha*. Finally, a neighbor-joining tree and Bayesian clustering analysis showed distinct clustering into two groups (*K* = 2), agreeing with the geographical distributions of these populations. Overall, our results will facilitate further studies of genetic diversity within *A. eriantha* and will aid in discriminating outlier loci involved in local adaptation.

## Introduction

*Actinidia eriantha* Benth. (Actinidiaceae) is a functionally dioecious, perennial woody vine (2*n* = 58) with a wide distribution in south central and south east China. The roots of *A. eriantha* have been used in traditional Chinese medicine to treat gastric carcinoma, nasopharyngeal carcinoma, breast carcinoma, and hepatitis (Sun et al., 2015). Polysaccharides isolated from roots have been shown to inhibit the growth of transplantable S180 sarcoma in mice, as well as promote splenocyte proliferation and natural killer cells activity (Xu et al., 2009).

*Actinidia* has also been recognized as a valuable species for commercial kiwifruit improvement. As an economically important kiwifruit cultivar produced by interspecific hybridization between *A. chinensis* (♀) and *A. eriantha* (♂), “Jinyan” has the greatest fruit weight, strongest fruit firmness, and longest shelf life compared to other commercial kiwifruit cultivars in China (Zhong et al., 2012). Moreover, the fruit of *A. eriantha* contains higher vitamin C concentrations (800 mg/100 g FW) than that of commercial cultivars of *A. chinensis* (85–110 mg ascorbate/100 g FW) (Huang et al., 1983; Seal, 2003). These traits can be used to improve the quality of commercial cultivars of *A. chinensis*.

Despite its potential in medicine and crop improvement, molecular studies on *A. eriantha* lag behind those of other important crop species, such as *Populus deltoides* Marshall (Fahrenkrog et al., 2017), *Ananas comosus* (L.) Merr. (Wai et al., 2016), and *Brassica napus* L. (Cao et al., 2016), due to a lack of genome-wide genetic resources. Genetic markers such as cpDNA (Cipriani et al., 1998; Li et al., 2007; Yao et al., 2015), mtDNA (Chat et al., 2004), nuclear SSR (Liu et al., 2010), and AFLP (Li et al., 2007) have been used to study the systematics, genetic diversity, and population structure of *Actinidia*. The most recent investigation into the genetic variation and population differentiation of *A. eriantha* used 9 nuclear microsatellite loci and six populations located in the border of Hunan Province and Guangxi Province (Liu et al., 2010). Consequently, more genetic resources are needed to develop an in-depth understanding of genetic diversity and population structure within *A. eriantha*.

Compared with selectively neutral markers, SSRs developed from expressed sequence tags (EST-SSRs) can reveal local adaptation and the effects of environmental heterogeneity since they are potentially tightly linked with functional genes controlling phenotype (Cordeiro et al., 2001; Varshney et al., 2005; Kumari et al., 2013; Chen et al., 2015). In addition, EST-SSR markers specifically designed for a single species frequently display a high degree of transferability to related species because of their location in conserved gene regions (Gupta et al., 2003; Pashley et al., 2006; Zheng et al., 2013; Guo et al., 2014). Improvements in high-throughput sequencing (HTS) technology have facilitated the sequencing of transcriptomes at a relatively low cost, enabling the rapid identification of EST-SSRs (Faure and Joly, 2015). The *de novo* assembly of transcriptomes in non-model organisms, especially when a sequenced genome is lacking, is essential for studying functional genomics or mining markers in these organisms (Loman et al., 2012; Faure and Joly, 2015).

In this study, we sequenced a fruit transcriptome from *A. eriantha* to first identify and characterize all unigenes present in the developing fruit. Second, we developed SSR markers and tested the transferability of the developed EST-SSRs to other related diploid species. Lastly, we demonstrate the usefulness of these markers by analyzing genetic diversity and structure of seven wild populations of *A. eriantha*.

## Materials and methods

### Plant material

Young fruits were collected from three individuals of *A. eriantha*, then immediately frozen in liquid nitrogen and stored at −80°C until RNA isolation. For EST-SSRs development, one individual from a wild population (YM, Mount Yangming) of *A. eriantha* was used to confirm the amplification specificity of the synthesized EST-SSR primers. Then, eight individuals from four wild populations (ShQ, YM, RY, and LiS) of *A. eriantha* were utilized for investigating levels of polymorphism in the SSR markers (Table 1). Lastly, 12 individuals from 10 additional *Actinidia* species were used to analyze the transferability of the polymorphic SSRs (Table 1). Finally, 186 individuals from seven wild populations of *A. eriantha* (ShQ, YM, RY, WH, RJ, LY, and LiS) sampled from South China and East China were used to assess levels of genetic diversity and population structure of extant populations (Table 1). The samples of *A. eriantha* used for RNA extraction and all samples of related species used for DNA extraction were collected from Wuhan Botanical Garden, Chinese Academy of Science in autumn 2014 and spring 2016, respectively. Other wild individuals of *A. eriantha* were sampled in summer 2015. Voucher specimens representative of all samples are stored at the Herbarium of Wuhan Botanical Garden. Fresh leaves were collected and desiccated in silica gel for DNA isolation.

**Table 1:** Sampling details for all species of *Actinidia* used in the present study.

**Species**	**Population code**	**Location**	**Latitude(N)**	**Longitude(E)**	**Altitude(m)**	**Sample size**
*Actinidia eriantha* Benth.	ShQ	Shiqian County, Guizhou Province	27°20′	108°09′	977	12
	YM	Mount Yangming, Hunan Province	26°08′	111°57′	1,179	15
	RY	Ruyuan County, Guangdong Province	24°57′	113°03′	820	33
	WH	Wuhua County, Guangdong Province	23°52′	115°23′	686	28
	RJ	Ruijin City, Jiangxi Province	25°56′	116°14′	400	30
	LY	Luoyuan County, Fujian Province	26°28′	119°25′	529	35
	LiS	Lishui City, Zhejiang Province	28°15′	119°47′	365	33
*Actinidia styracifolia* C. F. Liang	WZS	Wuhan Botanical Garden, Hubei province	30°33′	114°25′	28	1
*Actinidia zhejiangensis* C. F. Liang	WZZ	Wuhan Botanical Garden, Hubei province	30°33′	114°25′	28	2
*Actinidia latifolia* (Gardn. et Champ.) Merr.	WZL	Wuhan Botanical Garden, Hubei province	30°33′	114°25′	28	1
*Actinidia chinensis* Planch.	WZC	Wuhan Botanical Garden, Hubei province	30°33′	114°25′	28	1
*Actinidia kolomikta* (Maxim. et Rupr.) Maxim.	WZK	Wuhan Botanical Garden, Hubei province	30°33′	114°25′	28	1
*Actinidia hubeiensis* H. M. Sun & R. H. Huang	WZH	Wuhan Botanical Garden, Hubei province	30°33′	114°25′	28	2
*Actinidia valvata* Dunn	WZV	Wuhan Botanical Garden, Hubei province	30°33′	114°25′	28	1
*Actinidia rufa* (Siebold & Zuccarini) Planchon ex Miquel	WZR	Wuhan Botanical Garden, Hubei province	30°33′	114°25′	28	1
*Actinidia fulvicoma* Hance	WZF	Wuhan Botanical Garden, Hubei province	30°33′	114°25′	28	1
*Actinidia lijiangensis* C. F. Liang & Y. X. Lu	WZLi	Wuhan Botanical Garden, Hubei province	30°33′	114°25′	28	1

### RNA and DNA isolation

Total RNA was extracted from frozen fruits using TRIzol reagent (Invitrogen, Carlsbad, CA, USA) according to the manufacturer's instructions, followed by treating with RNase-free DNase I (Invitrogen, USA) for 30 min at 37°C to remove residual DNA. RNA was quantified using a NanoDrop 2000 (Thermo Fisher Scientific, Waltham, MA, USA) and validated by 1.5% agarose gel electrophoresis. Total genomic DNA was extracted from silica-dried leaves using a modified CTAB method (Doyle and Doyle, 1990). Quality and concentration of the DNA was confirmed using 1% agarose gel electrophoresis and NanoDrop 8000 (Thermo Fisher Scientific, Waltham, MA, USA).

### Transcriptome sequencing and *De novo* assembly

Messenger RNA was purified from 300 μg of total RNA using Dynabeads oligo(dT)25 (Thermo Fisher Scientific, Waltham, MA, USA) and subsequently used for cDNA library construction using the NEBNext Ultra™ RNA Library Prep Kit for Illumina (New England Biolabs, Beverly, MA, USA), following manufacturer's protocol. After library validation on a BioAnalyzer (Agilent 2100 Santa Clara, CA, USA), cDNA libraries were sequenced on a MiSeq (Illumina, Hayward, CA, USA) by Shanghai Hanyu Bio-Tech Co., Ltd (Shanghai, China).

Raw reads were filtered in Trimmomatic (version 0.30; Bolger et al., 2014). All reads exhibiting adaptor contamination, length less than 36 bp, and low quality scores (i.e., an average Q-value < 15) were removed. The cleaned reads were *de novo* assembled using TRINITY (Release-2012-06-08; Grabherr et al., 2011) with default parameters. After assembly, the TIGR Gene Indices clustering tools (TGICL) (version 2.1; Pertea et al., 2003) were used to cluster and remove redundant transcripts, and identify unigenes (i.e., non-redundant transcripts), which were used for subsequent analysis.

### Annotation and functional classification

Open reading frames (ORFs) were detected within unigenes using the “GetORF” program of EMBOSS (version 6.6.0; Rice et al., 2000). For each unigene, the longest ORF was predicted and unigenes with ORFs < 90 bp (30 amino acids) were discarded. The predicted protein-coding sequences were aligned against the public databases of NCBI non-redundant (NR) protein sequences, Gene Ontology (GO), Swiss-Prot, EuKaryotic Orthologous Groups of proteins (KOG), and Kyoto Encyclopedia of Genes and Genomes (KEGG) (E-value < 1e-5). The value with the highest matching alignment was considered to contain the annotation information.

GoPipe (version 2; Chen et al., 2005) was used to obtain Gene Ontology (GO) annotations of the unigenes after the predicted protein sequences were aligned with the Swiss-Prot and TrEMBL databases using BLASTp (under the threshold of 1e-5). Meanwhile, the KOG annotations were performed using BLASTp (E-value < 1e-3) through comparing the predicted protein sequences with the KOG database. To determine metabolic pathways, the predicted protein sequences were also mapped to the KEGG metabolic pathway database using the KEGG Automatic Annotation Server (KAAS) (Moriya et al., 2007) with reciprocal BLAST (E-value <1e-5). Thus, the KO number of the predicted protein was obtained, which allowed for information regarding the pathway related to the predicted protein to be acquired.

### Development and detection of EST-SSR markers and cross-species amplification

MIcroSAtellite (MISA) (http://pgrc.ipk-gatersleben.de/misa/) was used to mine the assembled fruit transcriptome for microsatellite markers. Candidate SSRs ranged from two to six nucleotides, with the minimum repeat unit defined as six repeats for dinucleotides and five repeats for all higher order motifs, following the method of Jurka and Pethiyagoda (1995). Adjacent EST-SSRs with interruptions less than 100 bases were defined as compound EST-SSRs. We analyzed the distribution of different nucleotide repeats occurring within the untranslated regions (UTRs) or ORFs in unigenes. To evaluate if there was a significant enrichment in GO categories between the full set of unigenes and these containing SSRs, we performed a GO analysis of the annotated SSR-containing unigenes.

Primer3 (Rozen and Skaletsky, 2000) was then used with default settings to design primers pairs that would generate PCR products ranging in size from 100 to 300 bp. Polymorphic maximization were used as the criteria to select the polymorphic loci. For maximizing the polymorphism, we selected SSR loci with a minimum of 10 repeats for dinucleotides, seven for trinucleotides, and five for tetranucleotides for amplification using a GeneAmp PCR System 9700 thermal cycler (Applied Biosystems, Foster City, USA). Reactions were conducted in a total volume of 10 μl containing 2 μl of genomic DNA (50 ng total), 0.25 units Taq DNA polymerase (TaKaRa, Dalian, Liaoning, China), 1 × PCR buffer, 1 μl of 2.5 mM MgCl_2_, 1 μl of 2.5 mM dNTPs, 0.1 μl bovine serum albumin (BSA) (TaKaRa, Dalian, Liaoning, China), and 0.5 μl of each 10 μM primer. Cycling conditions were 95°C for 5 min, followed by 35 cycles of 94°C for 45 s, 60°C for 1 min, 72°C for 1 min, and a final extension at 72°C for 10 min. The PCR products were run on 12% denaturing polyacrylamide gels using a 25 bp ladder (Promega Corporation, Madison, Wisconsin, USA) for reference and were visualized by silver staining.

### Population genetic analysis

To assess levels of genetic diversity and population structure of *A. eriantha* accurately, 14 polymorphic SSR loci were randomly selected. The 5′ end of the forward primer of each SSR was labeled with a fluorescent dye (6-FAM, HEX, or TAMRA), with PCR amplification conditions the same as described above. The products were separated on a 3730xl DNA Analyzer (Applied Bio-systems) with GeneScan 500 LIZ as an internal size standard and processed using GENEMARKER (version 2.2.0; SoftGenetics, Pennsylvania, USA). The number of observed alleles (*N*_*A*_), observed (*H*_*O*_), and expected (*H*_*E*_) heterozygosities, and polymorphism information content (PIC) values were calculated for each locus using CERVUS (version 3.0.3; Kalinowski et al., 2007). Deviation from Hardy-Weinberg equilibrium (HWE) was tested for all loci at the population level and across all populations using GENEPOP (version 4.2; http://genepop.curtin.edu.au/). To account for any departure from Hardy-Weinberg equilibrium due to the presence of null alleles (Soulsbury et al., 2007), the null allele frequency at each locus within each population was calculated using FreeNA (Chapuis and Estoup, 2007) following the Expectation Maximization (EM) method described by Dempster et al. (1977). Effects of null alleles were considered when the frequency of null alleles was higher than 0.2 (Dakin and Avise, 2004; Chapuis and Estoup, 2007). Potential loci under selection were tested using LOSITAN (Antao et al., 2008) with a 95% confidence level and 200,000 simulations following the method of Beaumont and Nichols (1996).

Genetic diversity and inbreeding parameters such as *N*_A_, *H*_E_, allele richness (*R*_S_, standardized for 12 individuals using rarefaction), and the average inbreeding coefficient (*F*_IS_) were estimated using FSTAT (version 2.9.3; Goudet, 2001) for each population using two loci sets: (i) all the EST-SSRs; and (ii) EST-SSRs excluding the loci with a high frequency of null alleles and/or effected by positive selection. We used GENEPOP to estimate the significance of departures from HWE, given by the deviations of *F*_IS_ values from zero. *F*_ST_ values and the significance (*p* < 0.05) between populations were estimated in ARLEQUIN (version 3.5; Excoffier and Lischer, 2010) with 10,000 permutations. Neighbor-joining (NJ) networks revealing the genetic relationships among populations were generated based on *D*_A_ distances (Takezaki and Nei, 1996) between populations using POPTREE (version 2; Takezaki et al., 2009). Bootstrap values were calculated from 100,000 replicates of resampled loci. Finally, a Bayesian clustering approach was used to assign individuals to genetic clusters using the software STRUCTURE (version 2.3.4; Pritchard et al., 2000; Falush et al., 2003). Assuming an admixture model and correlated allele frequencies, ten independent runs for each *K* value, ranging from 1 to 7, were performed with a burn-in of 50,000 generations and 500,000 total Markov chain Monte Carlo (MCMC) generations. To identify the most likely number of clusters (*K*), the rate of change of L(*K*) (Δ*K*) between successive *K* values was calculated following Evanno et al. (2005).

## Results

### Illumina sequencing and *De novo* assembly

**Figure 1:**
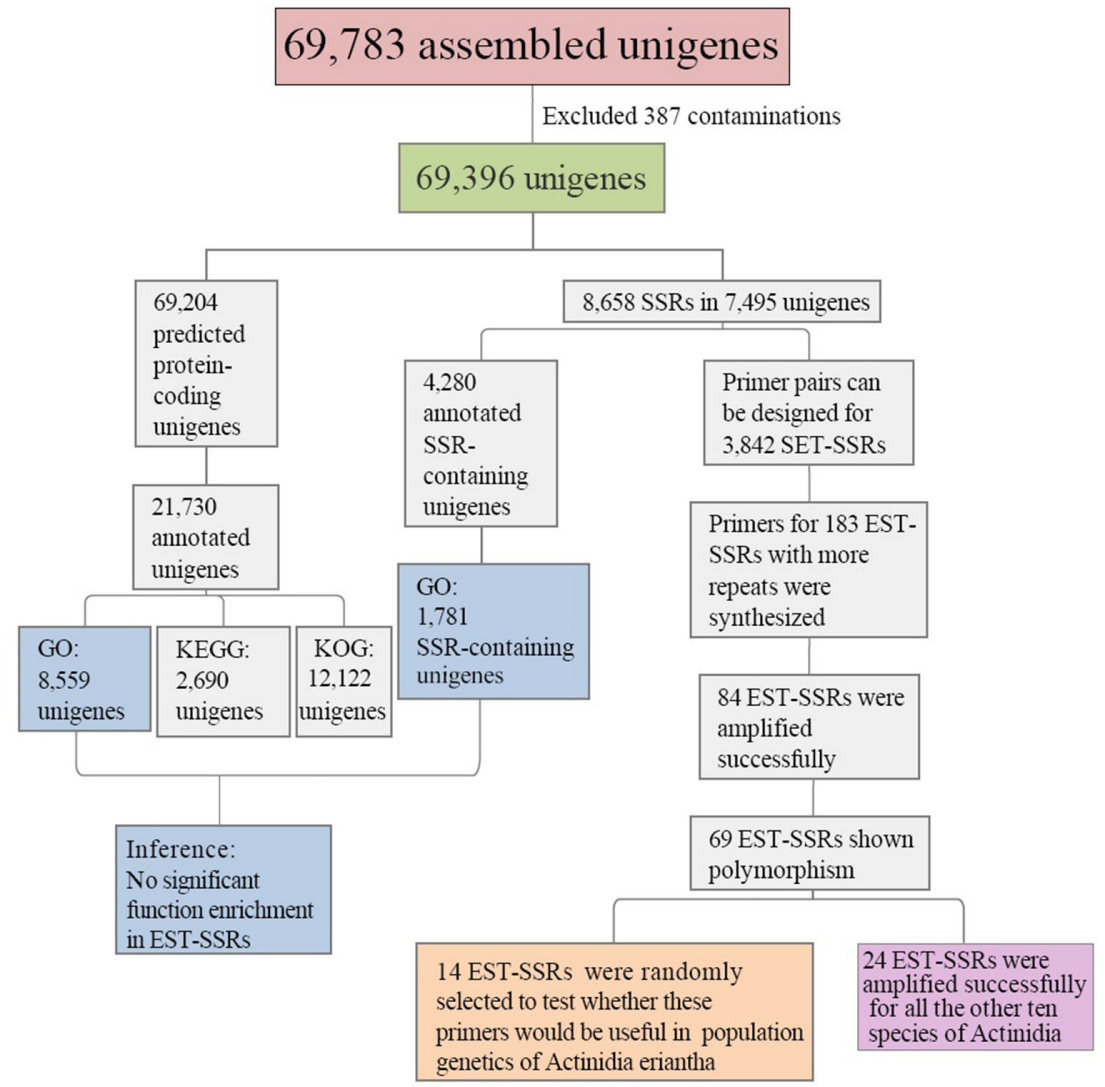
Outline of the process of unigene annotation and EST-SSR development in *Actinidia eriantha*.

**Figure 2:**
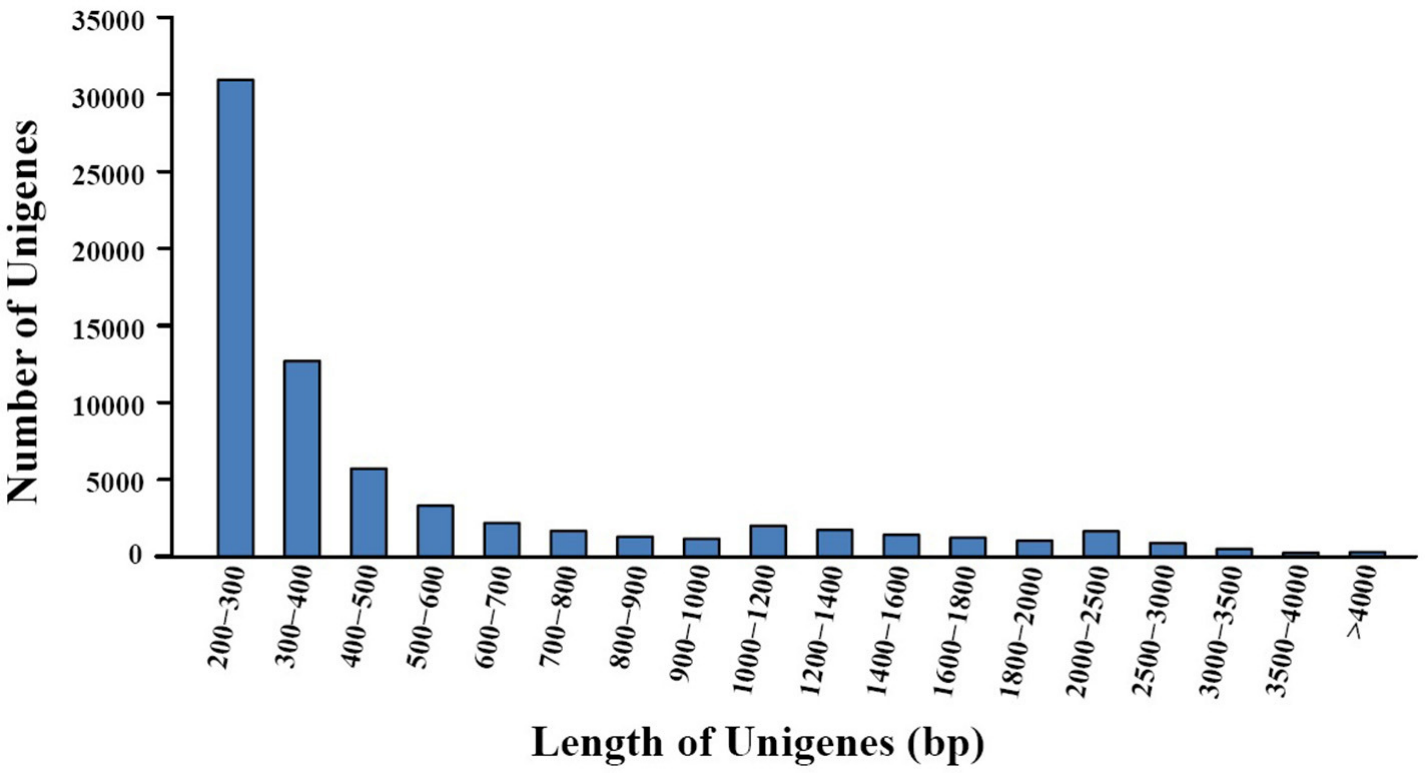
Distribution of unigene lengths resulting from *de novo* transcriptome assembly of fruits from *Actinidia eriantha*.

Transcriptome sequencing of *A. eriantha* produced 5,678,129 reads with a total output of 3.41 Gb (NCBI accession: SRR5565131). After data filtering, 5,677,503 (99.99%) reads were obtained, with zero ambiguous bases and a GC percentage of 45.90%. Assembly of filtered reads yielded a total of 69,783 non-redundant unigenes (41.3 Mb). A flow chart was made to summarize the process of unigene annotation and EST-SSR development of *A. eriantha* (Figure 1). According to the contamination report provided by NCBI Transcriptome Shotgun Assembly (TSA) Sequence Database, 387 contaminants were excluded. The other 69,396 unigenes (41.2 Mb) with lengths between 201 and 9,602 bp (Figure 2), a mean length of 594 bp, and an N_50_ of 973 bp, were retained for analyses.

### Functional annotation and metabolic pathway assignments

**Figure 3:**
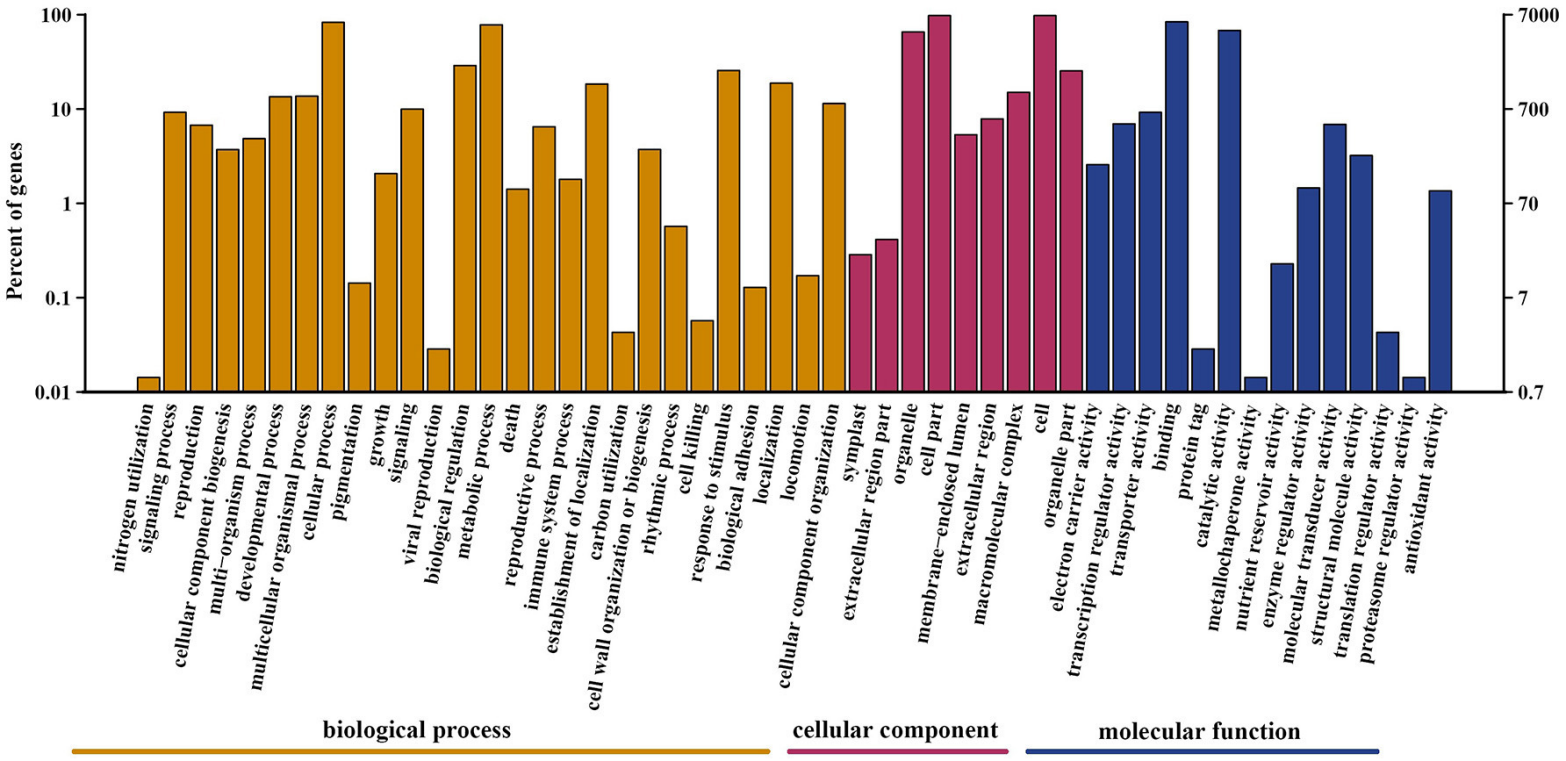
Gene Ontology classification of unigenes. Unigenes were assigned to three categories: cellular component, molecular function, and biological process.

A total of 69,204 protein-coding unigenes were predicted. Of these, 21,730 matched sequences in the public databases of NR, GO, Swiss-Prot, KOG, and KEGG (Table S1); whereas 47,474 were unannotated. Based on Swiss-Prot and TrEMBL annotation, 8,559 sequences from *A. eriantha* were categorized into the following three GO categories: biological process, cellular component and molecular function, with 3,387 functional terms, that were further subdivided into 50 subcategories (Figure 3). In the biological process category, 6,007 (70.2%) unigenes were classified under cellular process, and 5,603 (65.5%) unigenes were classified under metabolic process. In the cellular component category, cell (6,844; 80.0%) and cell part (6,844; 80.0%) were prominently represented. With respect to the molecular function category, binding (5,882; 68.7%) was the most abundant subcategory, followed by catalytic activity (4,769; 55.7%).

Unigenes were annotated and functionally classified into 25 KOG categories (Figure S1), and a large number of the unigenes were assigned to more than one category. Among these categories, signal transduction mechanisms (1,146), general function prediction only (1,016), and posttranslational modification, protein turnover and chaperone activity (902) were the three dominant groups.

**Figure 4:**
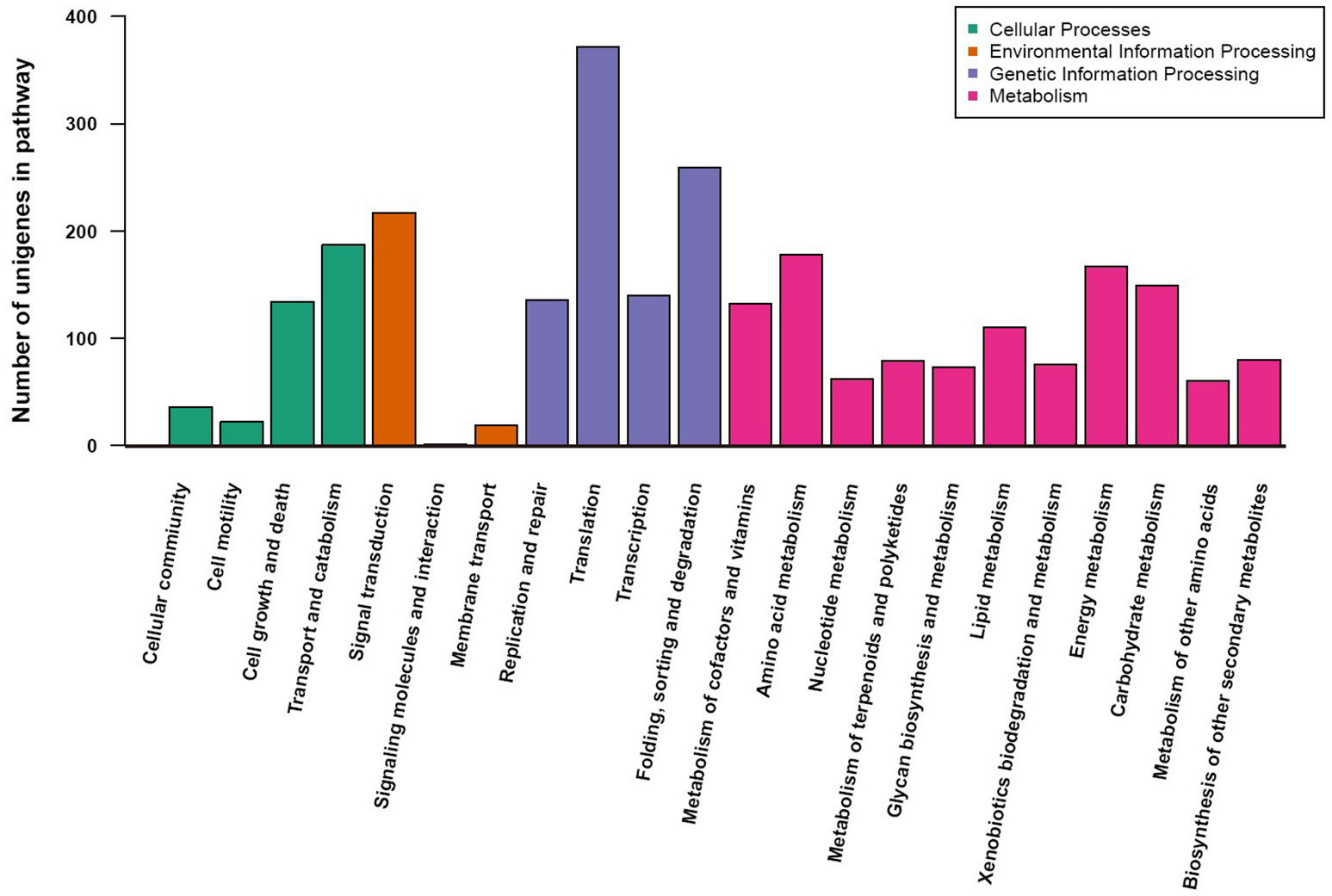
KEGG pathway assignment using a reciprocal BLAST analysis with a E-value cutoff of 1e–5.

To understand the metabolic pathways that might be active in the fruit of *A. eriantha*, the protein-coding sequences were compared against the KEGG database. A total of 2,690 unigenes were identified with pathway annotation, and they were functionally assigned to 22 KEGG pathways (Figure 4). The most highly represented pathways were Translation (372 unigenes; 13.8%), followed by Folding, sorting and degradation (259 unigenes; 9.6%), and Signal transduction (217 unigenes; 8.1%).

### Discovery, development, and transferability of EST-SSRs

Of the 69,396 non-redundant unigenes, a total of 8,658 EST-SSRs (including 409 compound SSRs) were identified in 7,495 unigene sequences, including 1,014 unigene sequences that contained more than one EST-SSR. The frequency of EST-SSRs in unigenes was 12.48%, and the distribution density of EST-SSRs was one per 4.76 kilobases (kb). The most common repeat motif was dinucleotide (7,084; 81.8%), followed by trinucleotide (1,428; 16.5%), tetranucleotide (134; 1.5%), and pentanucleotide (12; 0.1%), with 148 different motifs identified; no hexanucleotide repeats were identified. A total of 5,069 SSRs were present in UTRs and 3,586 in ORFs. Dinucleotide repeats were the most common SSR type in ORFs (2,616) and UTRs (4,465). Trinucleotide repeats occurred primarily in ORFs (927 in ORFs; 501 in UTRs), while other repeat motifs occurred mainly within UTRs (Table S2). Taking sequence complementarity into consideration, these motifs were reduced to 45 types (Table S3). The dominant motif type for di- to tetranucleotide repeat SSRs were AG/CT (6,069; 70.1%), AAG/CTT (402; 4.6%), and AAAT/ATTT (34; 0.39%), respectively. Twelve different types of pentanucleotide repeat SSRs were detected, each unique. EST-SSR repeat length ranged from 12 to 25 bp, with 12 bp being the most frequent (24.0%) (Figure S2). Of the 7,495 unigene sequences containing SSRs, 4,280 were annotated, and 1,781 unigenes were classified into 43 GO terms in three ontologies. The distribution of GO terms across the unigenes containing SSRs was similar to that across all 8,566 GO unigenes. Hence there was no significant enrichment in any GO categories for SSR-containing unigenes (data not shown).

Primer pairs were designed for 3,842 loci, representing 44.4% of all EST-SSR candidate loci (Table S4). Of these, primers for the 183 EST-SSR markers with 10 minimum repeat units for dinucleotide repeats, seven for trinucleotide repeats, and five for tetranucleotide repeats were synthesized and used for validation and assessment of polymorphism in *A. eriantha*. A total of 84 primer pairs (45.9%) exhibited successful amplification, of which 69 (37.7%) revealed polymorphism when screening with eight individuals from four populations. The number of alleles (*N*_*A*_) of those loci varied from two to eight, with a mean of 3.6 alleles per locus and a total of 251 alleles (Table S5).

Among the 69 polymorphic EST-SSR loci, 24 (34.8%) were amplified successfully across all tested species of *Actinidia*, while 37 amplified in some but not all species, and eight failed to amplify in all 10 additional species (Table S6). The four species with the highest success rates in cross-amplification trials were *Actinidia latifolia* (Gardn. et Champ.) Merr. (60, 87.0%), *A. styracifolia* C. F. Liang (59, 85.5%), *A. fulvicoma* Hance (58, 84.1%), and *A. zhejiangensis* C. F. Liang (58, 84.1%) (Figure S3).

### Population genetic diversity and structure

We detected a total of 85 alleles from the 14 loci used for assessing levels of genetic diversity and population structure of *A. eriantha* (Table 2), ranging from three alleles (locus AET-169) to 10 alleles (locus AET-144), with an average of 6.07 ± 2.30 alleles per locus. *H*_*O*_ ranged from 0.129 to 0.710 with a mean of 0.439, and *H*_*E*_ ranged from 0.229 to 0.822 with a mean of 0.619. PIC ranged from 0.216 to 0.797, with a mean of 0.579 (Table 2). Four loci (AET-38, AET-81, ATE-167, and AET-180) displayed significant deviation from HWE after Bonferroni adjustment (*p* < 0.0036) (Table 2). A high frequency of null alleles (frequency > 0.2) was observed at two loci: AET-38 for one population (LiS) and AET-167 for two populations (WH and RJ), suggesting that deviations from HWE at these loci can be mostly attributed to the presence of null alleles. Of the 14 loci, only AET-122 (outside the 95% confidence areas), which was identified in gene *comp29341_c0_seq5*, was affected by positive selection (Figure S4). This gene was annotated as F5O11.10 isoform 3 (NCBI No.: ref|XP_007039994.1|) which has been updated to PREDICTED: uncharacterized protein LOC18606366 isoform X1 (NCBI No.: ref|XP_007039994.2|).

**Table 2:** Fourteen EST-SSR loci used for assessing genetic differentiation of *Actinidia eriantha*

**Locus**	***N*_*A*_**	***H*_*O*_**	***H*_*E*_**	**PIC**	***p*-value of HWE**
AET-9	4	0.167	0.23	0.219	0.0093
AET-22	9	0.5	0.584	0.56	0.4745
AET-28	6	0.516	0.687	0.633	0.0127
AET-38	5	0.387	0.745	0.696	0
AET-81	8	0.468	0.727	0.681	0.0009
AET-82	4	0.285	0.363	0.334	0.799
AET-104	6	0.511	0.708	0.658	0.0113
AET-121	8	0.71	0.735	0.706	0.401
AET-122	4	0.5	0.702	0.641	0.6233
AET-141	9	0.597	0.822	0.797	0.1449
AET-144	10	0.672	0.782	0.745	0.8921
AET-167	5	0.317	0.739	0.691	0
AET-169	3	0.129	0.229	0.216	0.0082
AET-180	4	0.387	0.607	0.534	0

Based on the above results, genetic analyses were performed based on two sets of SSRs: (1) all 14 SSRs, and (2) the 11 SSRs remaining after excluding loci with high null alleles or under selection (AET-38, AET-167, and AET-122). In all cases described below, the first number refers to the 14 SSRs values, and the second number refers to the 11 SSRs values. Across the seven populations surveyed, population YM always possessed the lowest values for *N*_*A*_ (37/31), *R*_*S*_ (2.56/2.72), and mean *H*_*E*_ (0.329/0.366); while population RJ had the highest genetic diversity (*N*_*A*_ = 58/46; *R*_*S*_ = 3.69/3.69; mean *H*_*E*_ = 0.547/0.543) (Table 3). *F*_*IS*_ and *p*-values of HWE after Bonferroni correction based on all 14 SSRs revealed that four populations (ShQ, YM, RJ, and LiS) have significant heterozygote deficiency. After excluding the three loci due to null alleles or positive selection, only two populations (ShQ and LiS) were significantly deficient in heterozygote (Table 3).

**Table 3:** Population genetic diversity and inbreeding parameters for *Actinidia eriantha*.

**Population code (number of individuals)**	***N*_*A*_**	***R*_*S*_**	***H*_*E*_**	***F*_*IS*_**	***p*-value of HWE**
ShQ(12)	42/36	3.00/3.27	0.424/0.446	0.256/0.270	0.000/0.000
YM(15)	37/31	2.56/2.72	0.329/0.366	0.130/0.072	0.004/0.051
RY(33)	49/40	3.14/3.21	0.457/0.446	0.077/0.030	0.063/0.267
WH(28)	50/40	3.18/3.18	0.513/0.492	0.015/–0.016	0.098/0.139
RJ(30)	58/46	3.69/3.69	0.547/0.543	0.143/0.090	0.000/0.037
LY(35)	50/41	3.12/3.16	0.479/0.453	–0.048/–0.095	0.820/0.919
LiS(33)	56/45	3.48/3.44	0.505/0.464	0.168/0.079	0.000/0.000

*The first number in each pair represents values for all 14 SSRs, and the second number represents values for the 11 SSRs that do not have high null alleles and were not affected by positive selection*.

Overall, *F*_*ST*_ across the seven populations was 0.234/0.215, showing a high and significant genetic differentiation between populations. The pairwise *F*_*ST*_ estimates ranged from 0.094/0.086 (*p* < 0.05) between populations LY and LiS to 0.422/0.378 (*p* < 0.05) between populations YM and LY (Table 4). All the pairwise comparisons indicated significant differentiation between populations.

**Table 4:** Pairwise *F*_*ST*_ values of seven populations of *Actinidia eriantha*.

	**ShQ**	**YM**	**RY**	**WH**	**RJ**	**LY**	**LiS**
ShQ		–	–	–	–	–	–
YM	0.404/0.344		–	–	–	–	–
RY	0.344/0.305	0.291/0.287		–	–	–	–
WH	0.331/0.297	0.357/0.324	0.272/0.305		–	–	–
RJ	0.284/0.253	0.360/0.296	0.216/0.190	0.210/0.208		–	–
LY	0.336/0.332	0.422/0.378	0.290/0.254	0.276/0.261	0.148/0.110		–
LiS	0.309/0.312	0.343/0.321	0.214/0.210	0.237/0.236	0.112/0.087	0.094/0.086	

*The first number in each pair represents F_ST_ values for all 14 SSRs, and the second number represents F_ST_ values for the 11 SSRs that do not have high null alleles and were not affected by positive selection*.

**Figure 5:**
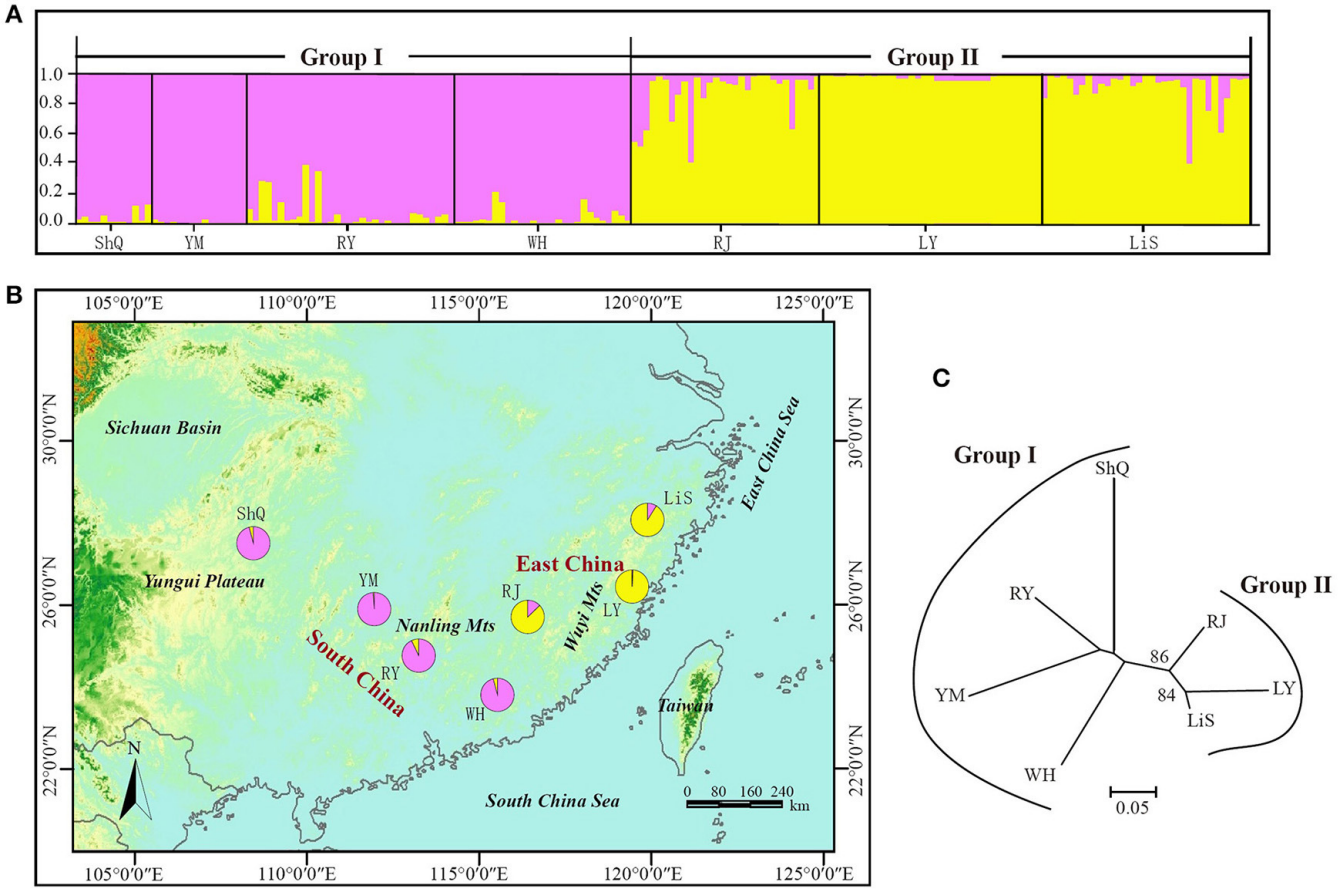
NJ tree and Bayesian clustering analysis results for 14 EST-SSRs of 186 individuals (7 populations) of *Actinidia eriantha* from South China and East China. **(A)** Histogram of the STRUCTURE analysis for the model with *K* = 2 (showing the highest delta K). Each individual is represented by a single vertical line. On the y-axes is the likelihood of assignment to each cluster. **(B)** Geographic origin of the seven populations and their color-coded grouping according to the STRUCTURE analysis. Population codes are identified in Table 1. **(C)** The neighbor joining tree of the seven populations with bootstrap values indicated in nodes with support (>50).

The NJ tree and Bayesian clustering analysis based on all loci indicated a high level of genetic structure (Figure 5). In the STRUCTURE analysis, the highest likelihood of the SSR data was obtained when samples were clustered into two groups (*K* = 2) (Figure S5). Group I included four wild populations (ShQ, YM, RY, and WH), and Group II contained populations RJ, LY, and LiS, in agreement with the geographical distributions of the sampled populations (Figure 5). The population-based NJ tree generally agreed with the output of STRUCTURE, although without significant bootstrap support. When the three loci most affected by null alleles or selection were discarded and the analysis rerun, the result was the same as that of 14 EST-SSRs (data not shown).

## Discussion

### Illumina sequencing and gene functional annotation/classification

Transcriptome sequencing of *A. eriantha* fruits yielded ~5.7 million high-quality reads that were assembled into 69,396 non-redundant clean unigenes (41.2 Mb) with a mean length of 594 bp. Similar results have been reported in previous published plant transcriptome studies of *Cinnamomum camphora* (L.) Presl (mean length 584 bp; Jiang et al., 2014), *Eleocharis dulcis* (N. L. Burman) Trinius ex Henschel (mean length 617 bp; Liu et al., 2015), and *Lindera glauca* (Sieb. et Zucc.) Bl. (mean length 560 bp; Niu et al., 2015). This suggests that the transcriptome sequencing data of *A. eriantha* was effectively assembled in the present study, which was further validated by the PCR amplification rate of the EST-SSR markers.

Of the 69,396 unigenes, 21,730 (31.3%) had homologs in the public protein databases of NR, GO, Swiss-Prot, KOG, and KEGG. The large number of unigene annotations could provide valuable information for future studies on *A. eriantha* such as guiding future detailed studies on metabolic pathways. The large number of unannotated sequences in *A. eriantha* is similar to that documented for other non-model organisms (Wang et al., 2014; Wu H. et al., 2014; Castro et al., 2015; Chen et al., 2015). Among the unannotated 47,666 unigenes, 192 were not predicted as protein-coding sequences, and these likely correspond to non-coding RNAs. The remaining 47,474 unannotated protein-coding sequences may represent incomplete sequences lacking informative domains for conclusive annotation and/or novel genes specific to *A. eriantha* that have not been previously characterized; they may also represent orphan enzymes (known enzyme activities lacking an associated protein sequence) (Carlsson, 2008; Sorokina et al., 2014). Annotation error in public protein databases (e.g., Swiss-Prot, NR, and KEGG) may also contribute to unannotated unigenes (Schnoes et al., 2009).

### Characterization of EST-SSRs

In this study, 8,658 EST-SSRs were discovered in 7,495 of a total of 69,396 unigenes. The EST-SSR distribution density was one SSR per 4.76 kb which is higher than in Triticeae (1/5.4 kb; Peng et al., 2005), *Luffa acutangula* (L.) Roxb. (1/8.06 kb; Wu H. B. et al., 2014), *Chrysanthemum indicum* Linnaeus (1/14.7 kb; Wang et al., 2013), *Pinus fenzeliana* Hand.-Mzt. var. *dabeshanensis* (W. C. Cheng & Y. W. Law) L. K. Fu & Nan Li (1/23.08 kb; Xiang et al., 2015) but lower than in *Dysosma versipellis* (Hance) M. Cheng ex Ying (1/2.46 kb; Guo et al., 2014) and *Hevea brasiliensis* (Willd. ex A. Juss.) Muell. Arg. (1/281.39 bp; Li et al., 2012). However, the distribution density of EST-SSRs is affected by a number of factors including SSR search criteria, SSR development tools, and the size of the database (Varshney et al., 2005; Parchman et al., 2010).

In *A. eriantha*, dinucleotide (81.8%) and trinucleotide repeats (16.5%) were the most dominant SSR repeat types, with higher repeat motifs being much rarer. Similar results have been reported in the transcriptomes of *Myrciaria dubia* (Kunth) McVaugh (Carlsson, 2008) and *L. acutangula* (L.) Roxb. (Wu H. B. et al., 2014). However, trinucleotide (16.5%) repeats have been observed to have the highest frequency in many other species such as *Vigna mungo* (L.) Hepper (Souframanien and Reddy, 2015), *Vigna radiata* (L.) Wilczek (Gupta et al., 2014), *Phaseolus vulgaris* L. (Blair et al., 2011), and *D. versipellis* (Guo et al., 2014). Qiu et al. (2010) demonstrated that the distribution of di-, tetra-, and pentanucleotide repeats occur mainly within the untranslated regions (UTRs), while tri- and hexanucleotide repeats occur primarily in exons. Our results are largely consistent with these observations (Table S2). However, the dominance of dinucleotide repeats in the present study is not due to the over- representation of UTRs compared with open reading frames, as proposed by Kumpatla and Mukhopadhyay (2005). Regardless of ORFs or UTRs, the proportion of dinucleotide was always the highest SSR type for *A. eriantha*.

Of the 45 motif types, the dominant motif type among dinucleotide repeats was AG/CT (6,069; 70.1%). Previous studies on monocots [e.g., *Colocasia esculenta* (L.) Schoot; You et al., 2015] and eudicots (e.g., *D. versipellis*; Guo et al., 2014) also indicated that AG/CT was the most abundant dinucleotide repeat motif. The AG/CT motif can represent GAG, AGA, UCU, and CUC codons, which translate into the amino acids Arg, Glu, Ala, and Leu, respectively. The relatively high frequency of Ala and Leu over other amino acids (Kantety et al., 2002; Qiu et al., 2010) may help explain why AG/CT motifs are present at such high frequency in plants (Morgante et al., 2002; Varshney et al., 2002). Of the trinucleotide motifs, AAG/CTT (402; 4.6%) was the most common. Previous studies on other species have shown that this motif is prominent in the transcriptomes of eudicots [*M. dubia*, (Carlsson, 2008); *D. versipellis*, (Guo et al., 2014); *Neolitsea sericea* (Bl.) Koidz., (Chen et al., 2015)]. This may result from the fact that this triplet codes for Lys, which is commonly found in the exons of plants (Katti et al., 2001; Li et al., 2004). In contrast, CCG/CGG (84; 1.0%) was a rare motif type in the transcriptome of *A. eriantha*. This is similar to other eudicots [*D. versipellis*, (Guo et al., 2014); *Epimedium sagittatum* (Sieb. et Zucc.) Maxim, (Zeng et al., 2010); *Raphanus sativus* L., (Jiang et al., 2012)] but markedly different from monocots, where this motif is among the most abundant [*C. esculenta*, (You et al., 2015); *Hordeum vulgare* L., *Zea mays* L., *Oryza sativa* L., and *Sorghum bicolor* (L.) Moench, (Kantety et al., 2002)]. This difference has been attributed to the high GC content of monocot (especially grass) genomes (Morgante et al., 2002).

### Polymorphism and cross-species transferability of EST-SSRs

Primer pairs were successfully designed for 3,842 loci (44.4%) of the 8,658 EST-SSR candidate loci using PRIMER3 (Table S4). Primer design for the remaining loci failed due to short flanking sequences of the SSR loci or inappropriate repeats for desired SSR markers. Of the 183 primer pairs that satisfied our design criteria, 84 (45.9%) were successfully amplified across eight individuals from the four populations of *A. eriantha*, with 69 (37.7%) being polymorphic. This rate of polymorphism is lower than that in *L. acutangula* (40.7%; *n* = 42) (Wu H. B. et al., 2014) and *V. mungo* (58.2%; *n* = 18) (Souframanien and Reddy, 2015), but higher than that in *Rosa roxburghii* Tratt. (29.4%; *n* = 16) (Yan et al., 2015) and *D. versipellis* (23.8%; *n* = 12) (Guo et al., 2014). A total of 251 alleles were detected in these polymorphic loci with a mean of 3.6 alleles per locus (Table S5). The polymorphic level of markers was correlated with the genetic diversity and number of materials tested (Wu H. B. et al., 2014). Only eight (11.6%) of the 69 polymorphic EST-SSR loci could not be amplified successfully from the other 10 *Actinidia* species (Table S6). This high transferability confirms that the flanking sequences of the EST-SSRs possess a high level of conservation among closely related species (Aggarwal et al., 2007; Wu J. et al., 2014). *Actinidia latifolia, A. styracifolia, A. fulvicoma*, and *A. zhejiangensis* exhibited the highest success rates in cross-amplification trials (Figure S3). This result is not surprising because of their close phylogenetic relationship with *A. eriantha*; previous studies have shown that *A. latifolia* and *A. styracifolia* are the closest paternal relatives of *A. eriantha* (Chat et al., 2004; Li et al., 2007; Yao and Huang, 2016), whereas *A. zhejiangensis* is a close maternal relative of *A. eriantha* (Li et al., 2007; Yao and Huang, 2016). In addition, *A. fulvicoma* is also a close relative based on a UPGMA tree constructed from AFLP loci (Li et al., 2007).

### Population genetic diversity and structure within *Actinidia eriantha*

EST-SSR polymorphism levels within *A. eriantha* in this study were lower (mean *H*_*E*_ = 0.619) than for SSRs developed from genomic DNA using traditional methods (mean *H*_*E*_ = 0.728) (Liu et al., 2010). This lower level of allelic diversity at EST-SSRs is most likely due to functional constraints in transcribed regions of the genome (Ellis and Burke, 2007), or to the type and number of repeat units and the gene region in which they occur (Bouck and Vision, 2007). However, compared with EST-SSR based studies in other plants including *V. mungo* (mean PIC = 0.26) (Souframanien and Reddy, 2015), *Pinus dabeshanensis* (mean PIC = 0.38) (Xiang et al., 2015), and *N. sericea* (mean PIC = 0.47) (Chen et al., 2015), polymorphism levels in this study were higher (mean PIC = 0.579), indicating that the EST-SSRs developed from *A. eriantha* are quite useful for revealing the genetic diversity of the species in further research.

Genetic analyses based on all 14 SSRs and the reduced subset of 11 SSRs indicated that estimates of *F*_IS_ and HWE were seriously affected by the three loci displaying significant numbers of null alleles or effects of selection (AET-38, AET-122, and AET-167) (Table 3), whereas population structure was only slightly affected (Figure 4) (see also Chapuis and Estoup, 2007; Carlsson, 2008). This reinforces the need to carefully assess loci for violation of neutral evolution when selecting loci for studies of population-level variation.

We detected significant differentiation among populations of *A. eriantha* (*F*_ST_ = 0.234/0.215; *P* = 0), in contrast to the much lower differentiation (*F*_ST_ = 0.155) detected for this species by Liu et al. (2010). This lower level of differentiation is most likely due to their sampling at the border of Guangxi Province and Hunan Province, whereas our samples were from a much wider geographic distribution (South China and East China). The NJ tree and Bayesian clustering analysis clearly clustered the *A. eriantha* genotypes into two separate groups, with most of the genotypes grouped according to their geographical origin (Figure 5). This phylogeographic structure suggests that populations from widely distinctive geographic regions of the range of *A. eriantha* may possess important differences in germplasm that may be useful for crop improvement efforts and may be important for conservation. Furthermore, our ability to detect this structure demonstrates that our newly developed EST-SSR markers will be useful for dissecting evolutionary processes that have shaped the genetic structure of *A. eriantha*. More specifically, these EST-SSRs will be used in combination with cpDNA markers to determine whether phylogeographic population structure has been shaped by vicariance and/or local adaptation in wild populations of *A. eriantha*.

## Conclusions

The results presented here promote our current understanding of the molecular biology of *A. eriantha*. The EST-SSR markers generated in this study will also facilitate further studies of genetic diversity and differentiation of *A. eriantha* and will aid in understanding possible local adaptation. In addition, these markers can be used for population genetics studies of other related species due to their high transferability.

## Data archiving statement

Raw Illumina reads: the NCBI accession is SRR5565131. It will be released in October 16, 2017. All primer pairs of the SSR markers are provided in Table S4.

## Author contributions

RG performed the experimental work and the statistical analysis, and contributed to drafting the manuscript. JL and MM contributed to data interpretation and assisted in drafting the manuscript. AM assisted with field manipulation and natural population handling. SJ contributed to the statistical analysis. XY and HW conceptualized and coordinated the project and assisted in drafting the manuscript. All authors read and approved the final manuscript.

### Conflict of interest statement

The authors declare that the research was conducted in the absence of any commercial or financial relationships that could be construed as a potential conflict of interest.
