# Supplementary material for: Development and Application of Transcriptome-Derived Microsatellites in Actinidia eriantha (Actinidiaceae)
Source: Front Plant Sci. 2017 Aug 25;8:1383. doi: 10.3389/fpls.2017.01383 (PMC5574902; doi:10.3389/fpls.2017.01383)
Supplement: Supplementary file 7 [file Presentation1.ZIP › Figure S1 KOG.pdf]

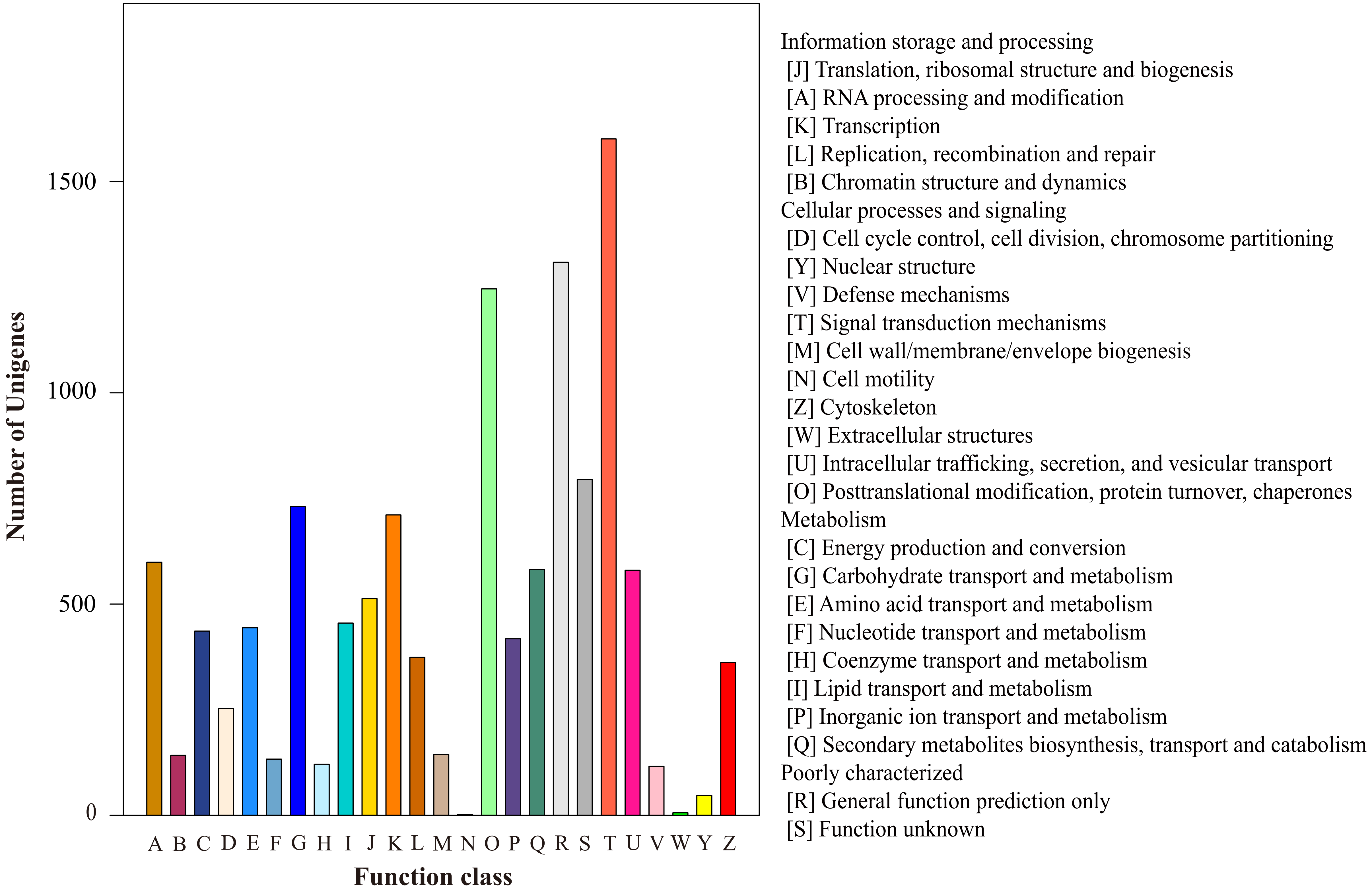

Figure S1 EuKaryotic Orthologous Groups of proteins (KOG) functional classification of A*ctinidia eriantha* Transcriptome sequences. 12,122 Unigenes were classified to 25 KOG categories.
