# Supplementary material for: Development and Application of Transcriptome-Derived Microsatellites in Actinidia eriantha (Actinidiaceae)
Source: Front Plant Sci. 2017 Aug 25;8:1383. doi: 10.3389/fpls.2017.01383 (PMC5574902; doi:10.3389/fpls.2017.01383)
Supplement: Supplementary file 7 [file Presentation1.ZIP › Figure S2 the length of SSRs.pdf]

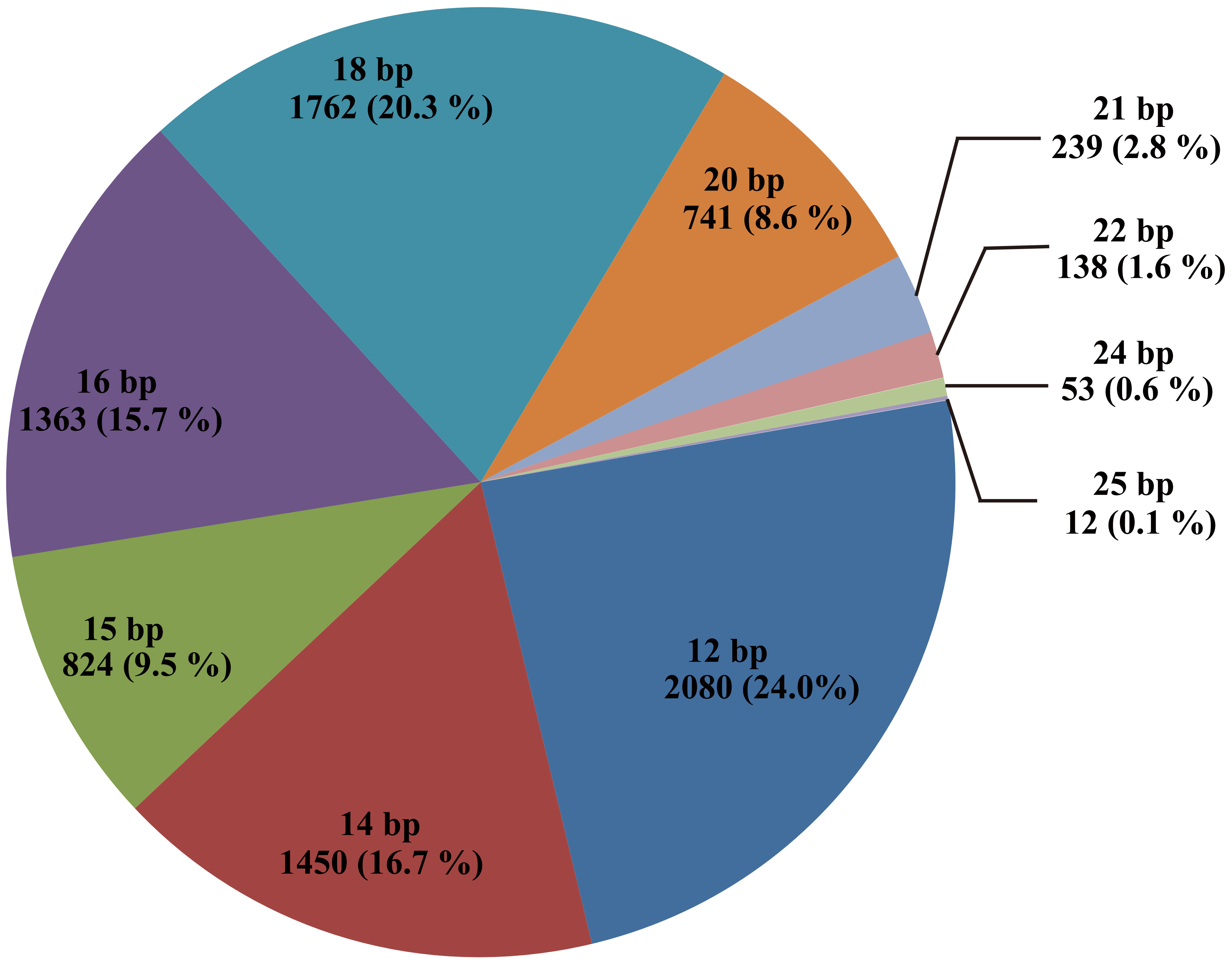

Figure S2 Length frequencies of EST-SSRs discovered from transcriptome sequences of *Actinidia eriantha* fruits.
