# Supplementary figures and images for: Development and Application of Transcriptome-Derived Microsatellites in Actinidia eriantha (Actinidiaceae)
Source: Front Plant Sci. 2017 Aug 25;8:1383. doi: 10.3389/fpls.2017.01383 (PMC5574902; doi:10.3389/fpls.2017.01383)

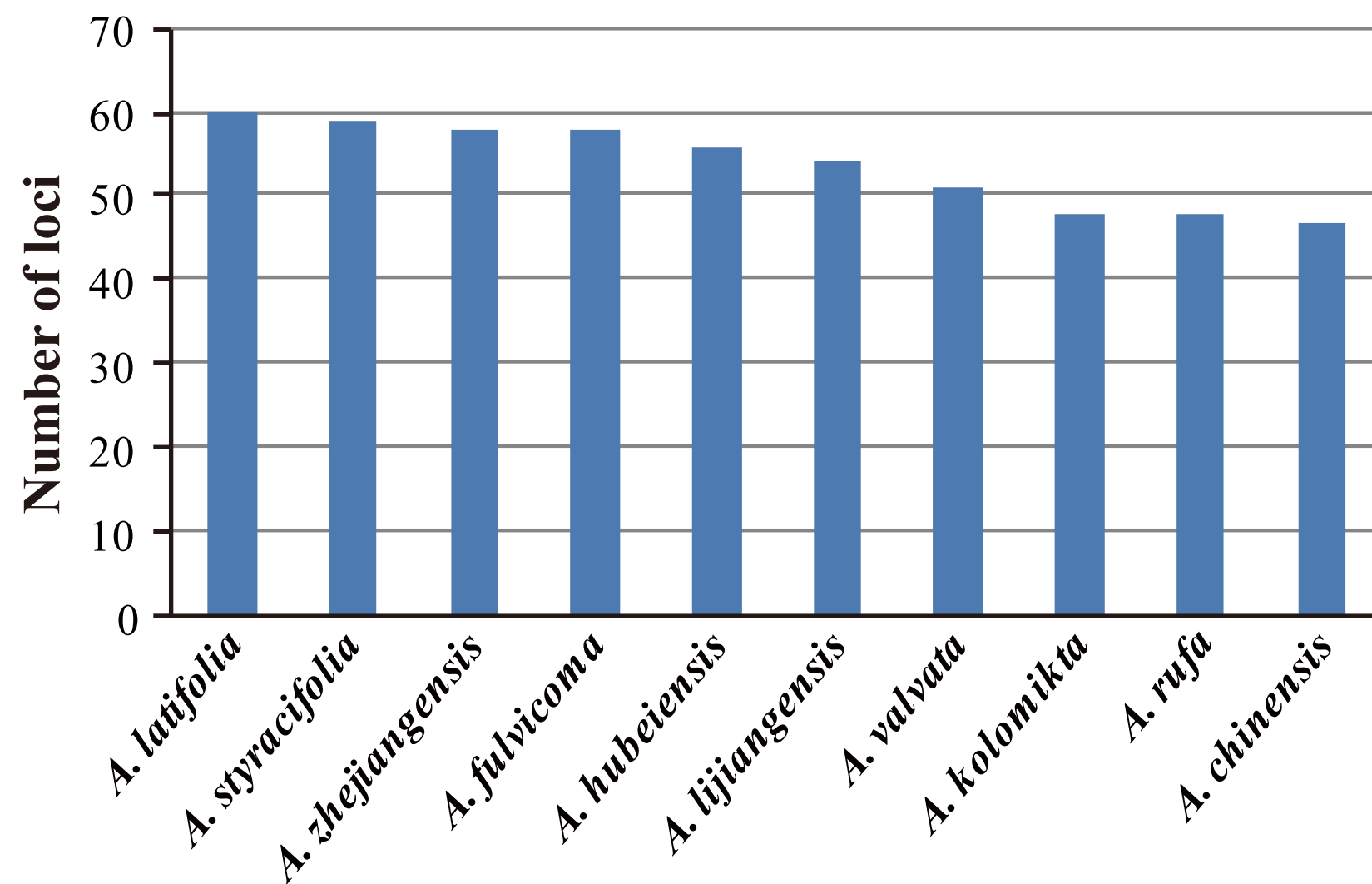

**Figure S3** The number of loci transferable across related species.

Supplement: Supplementary file 7 [file Presentation1.ZIP › Figure S3 The transferability.pdf]

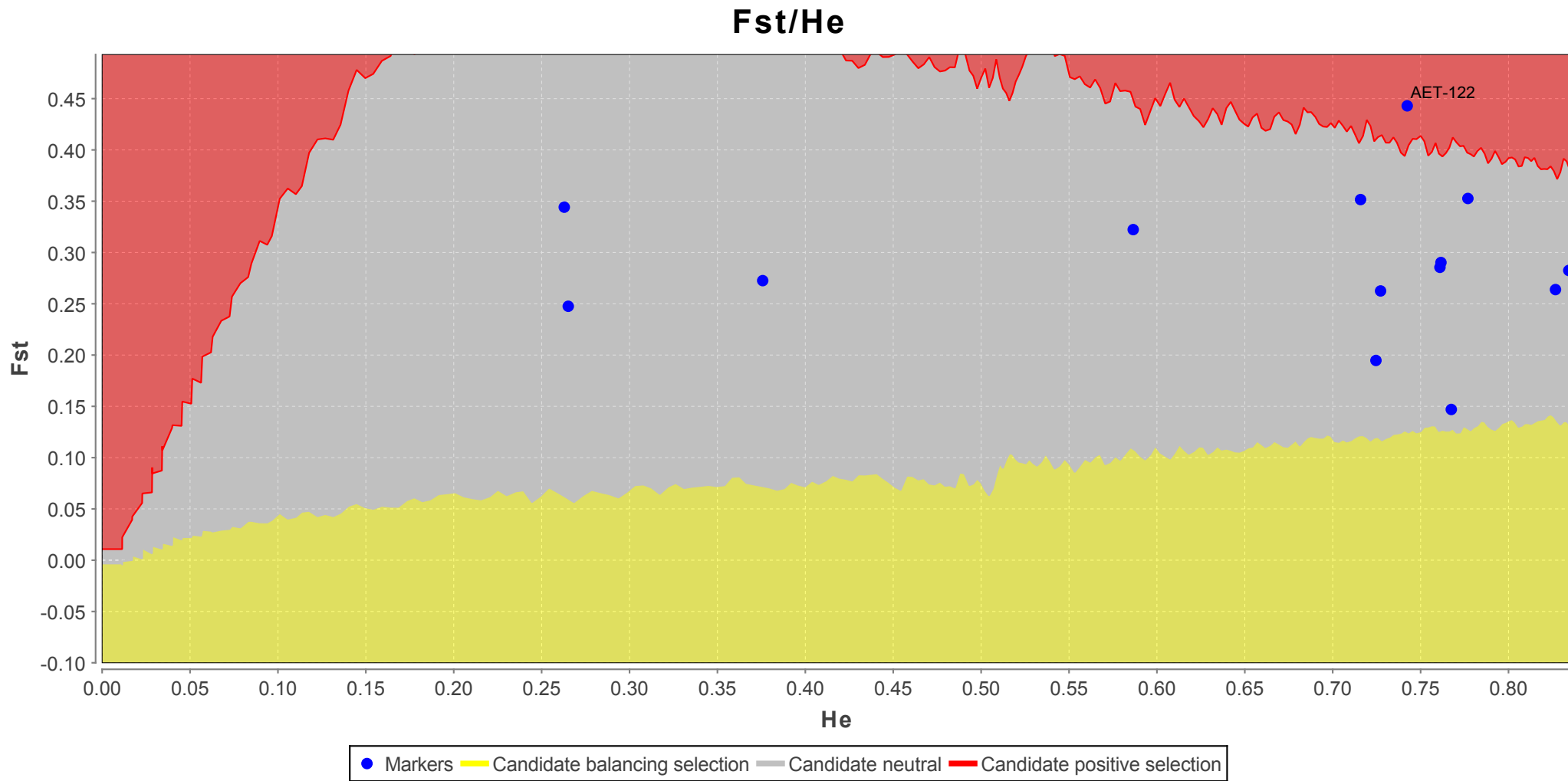

**Figure S4** Graphical result from LOSITAN for 14 EST-SSRs.

Supplement: Supplementary file 7 [file Presentation1.ZIP › Figure S4 LOSITAN result.pdf]

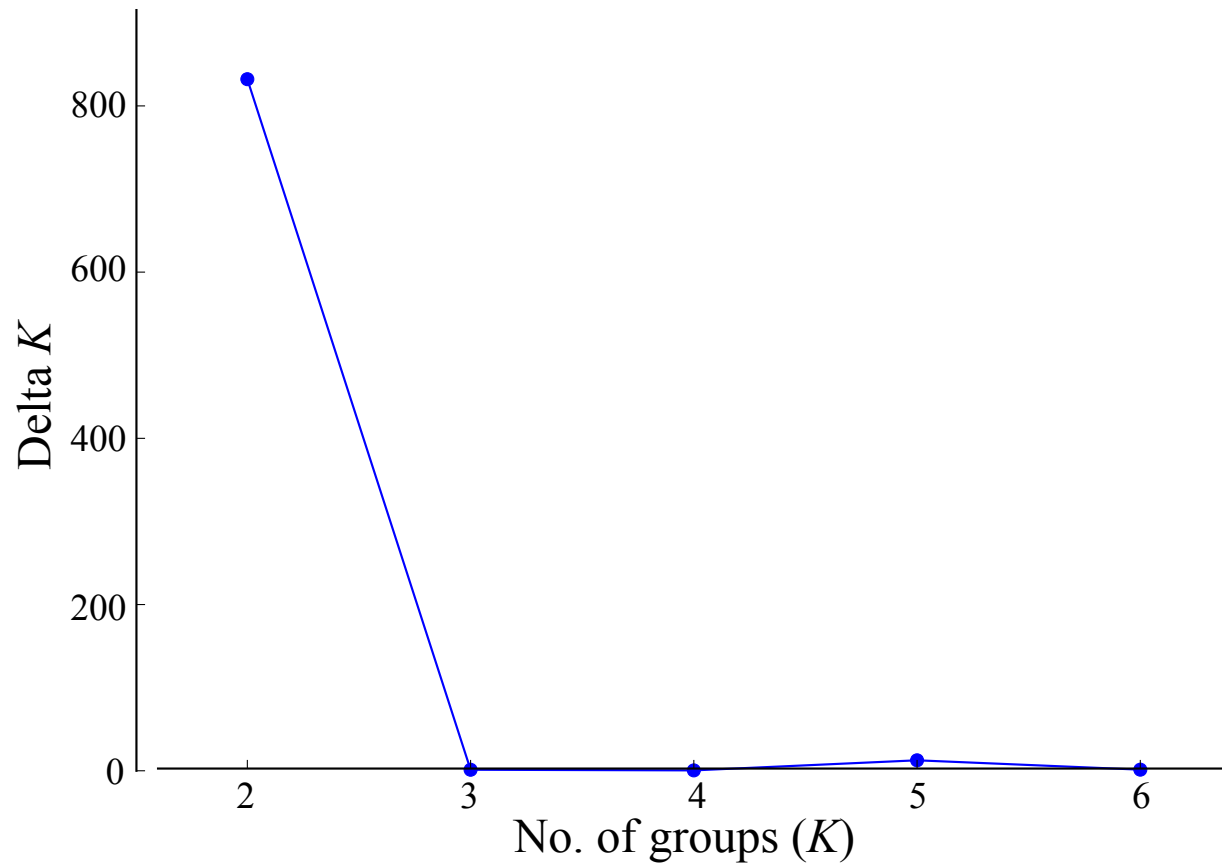

**Figure S5** Delta  $K$  statistics calculated according to Evanno et al. (2005) of each  $K$ .

Supplement: Supplementary file 7 [file Presentation1.ZIP › Figure S5 Delta K.pdf]
